# Implementing Gait Kinematic Trajectory Forecasting Models on an Embedded System

**DOI:** 10.3390/s24082649

**Published:** 2024-04-21

**Authors:** Madina Shayne, Leonardo A. Molina, Bin Hu, Taylor Chomiak

**Affiliations:** 1 Department of Mechanical and Manufacturing Engineering, University of Calgary, 2500 University Drive NW, Calgary, AB T2N 1N4, Canada; 2 CSM Optogenetics Platform, University of Calgary, 3330 Hospital Drive, Calgary, AB T2N 4N1, Canada; leonardo.molina@ucalgary.ca; 3 Division of Translational Neuroscience, Department of Clinical Neurosciences, Hotchkiss Brain Institute, Alberta Children’s Hospital Research Institute, Cumming School of Medicine, University of Calgary, 3330 Hospital Drive NW, Calgary, AB T2N 4N1, Canada; hub@ucalgary.ca

**Keywords:** gait, sensor, wearable, embedded system, forecast

## Abstract

Smart algorithms for gait kinematic motion prediction in wearable assistive devices including prostheses, bionics, and exoskeletons can ensure safer and more effective device functionality. Although embedded systems can support the use of smart algorithms, there are important limitations associated with computational load. This poses a tangible barrier for models with increased complexity that demand substantial computational resources for superior performance. Forecasting through Recurrent Topology (FReT) represents a computationally lightweight time-series data forecasting algorithm with the ability to update and adapt to the input data structure that can predict complex dynamics. Here, we deployed FReT on an embedded system and evaluated its accuracy, computational time, and precision to forecast gait kinematics from lower-limb motion sensor data from fifteen subjects. FReT was compared to pretrained hyperparameter-optimized NNET and deep-NNET (D-NNET) model architectures, both with static model weight parameters and iteratively updated model weight parameters to enable adaptability to evolving data structures. We found that FReT was not only more accurate than all the network models, reducing the normalized root-mean-square error by almost half on average, but that it also provided the best balance between accuracy, computational time, and precision when considering the combination of these performance variables. The proposed FReT framework on an embedded system, with its improved performance, represents an important step towards the development of new sensor-aided technologies for assistive ambulatory devices.

## 1. Introduction

The advancement in sensor-aided gait kinematic forecasting methods that can incorporate ongoing user-specific sensor signal information on an embedded system could have a significant impact on the design of intelligent lower-limb assistive devices [1]. Unlike fixed-motion capture systems, embedded systems offer a low-cost approach for the collection of high-quality, multi-source data for motion control purposes [2,3]. Moreover, the portability of wearable embedded systems has numerous practical applications for human–robot interactions to enable more efficient integration of a user’s natural body motion with an assistive device [4,5]. For instance, the deployment of sensor-aided gait kinematic forecasting technologies on embedded systems can be used to help reduce damage to joints, enhance rehabilitation outcomes, and even reduce the healthcare economic burden by lowering total healthcare system utilization [4,5]. While embedded systems can support the use of gait kinematic forecasting models for a variety of integrated robotic technologies, there are important limitations related to computational load when using complex state-of-the-art models on embedded systems [1]. To address this, the primary objective of this study was to deploy and evaluate a new computationally lightweight prediction algorithm with reduced complexity and demand for substantial computational resources to enable more effective gait kinematic forecasting on an embedded system. In the following, we will first outline work specifically related to this topic and then describe the methodology and experimental results that are discussed in relation to the use of this new algorithm for sensor-aided gait kinematic forecasting on embedded systems.

## 2. Related Work

Walking is an essential activity in daily life. Mobility augmentation through the use of wearable assistive ambulatory devices can provide vertical support, assist in lower-limb motion, and improve the quality of life for users [4,6,7,8,9,10,11,12,13,14,15]. In addition to the medical applications of wearable assistive ambulatory devices, the impact of various non-medical applications of these devices is also being realized, as they may be able to help healthy individuals perform important activities in daily life [4,5,16]. While there are intricate complexities related to safety regulatory requirements, user acceptance, as well as device reliability and adaptability that need to be considered, assistive ambulatory devices have the potential to help in such situations and may even reduce the burden on healthcare resources [4]. Nevertheless, given the importance of mobility assistance for both medical and non-medical end-user applications, there has been increasing interest in designing more effective and intelligent assistive ambulatory technologies [1,4,5,6,8,9,11,12,17,18,19,20,21,22,23,24,25,26,27,28,29].

Walking trajectory-tracking controllers, characterized by the sensor-aided forecasting of gait kinematic trajectories, represent the next generation in the design of assistive ambulatory devices. This technology aims to achieve natural, stable, and accurate interactive control with respect to human motion intention [3]. It can be used to reduce injury to users as well as compensate for delays in the response time of more complex control systems [1,3,17,18,22,23,30]. Forecasting gait kinematics can also enable targeted functional electrical stimulation at specific points in the gait cycle, leading to more effective rehabilitation therapy, and may even minimize the risk of falling by detecting deviations in the anticipated gait trajectory [1,23,28,29]. However, while lower-limb kinematic trajectory prediction can be used to solve numerous problems facing lower-limb robotics, a significant limiting factor for the implementation of accurate gait forecasting in the design of smart devices is the inability of most forecasting models to support continuous model learning [1]. Continuous learning (CL) would enable adaptive model building to continuously incorporate user-specific [25,31] and current dynamic signal information. This can lead to reliable predictions needed for improved device functionality and user safety [8,10,17,24] while avoiding prediction errors when pretrained models are used under unexpected conditions or conditions that were not included in the initial training process [32,33,34]. This is particularly relevant for gait, which is dynamically modulated to adjust for differing environmental conditions and meet the needs of ever-changing motor demands [26].

An obvious prerequisite for adaptive model building is the ability for CL to be completed quickly. However, modern prediction models are inherently complex, often requiring hyperparameter optimization and tuning in high-dimensional parameter space [1,6,35,36,37,38]. Increasing the number of parameter weights, which is inextricably linked with model architecture complexity, also increases computation time [39]. While increasing model complexity can improve model performance, it is at the expense of rapid CL capabilities [1]. In other words, the fast arrival of new data, together with increasingly slow retraining and optimization processes that are constrained on embedded ambulatory systems, poses a significant barrier to CL. Thus, new methods that permit CL capabilities on embedded systems could have a broad range of practical applications.

Local topological recurrence analysis represents a new computational approach we developed for identifying emergent recurring patterns in a signal’s surface topology [40]. Recently, we have expanded on the theoretical and practical properties of local topological recurrence analysis to provide a simple solution for time-series forecasting [38]. Forecasting through Recurrent Topology (FReT) identifies recurring patterns in local signal shapes to reveal unique memory traces embedded in a time-series signal that can be used to forecast a system’s upcoming time evolution [38]. Furthermore, unlike many other types of models, there is no need for computationally costly optimization and tuning procedures [38]. FReT’s simplicity may therefore enable new capabilities for CL to incorporate current dynamic signal information into forecasting. Hence, the objective of this study was to deploy and evaluate FReT-enabled gait kinematic forecasting on an embedded system for prototyping on affordable, embedded hardware that can simplify the integration of sensor-aided gait forecasting into real-world applications.

## 3. Materials and Methods

### 3.1. Artificial Neural Network Models

**Figure 1:**
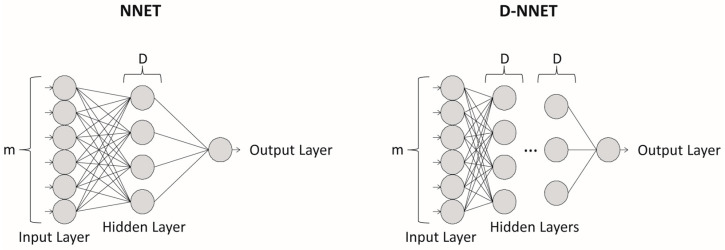
Basic artificial neural network architecture for time-series forecasting. The widely used NNET model (**left**), given its versatility in modelling a wide range of time-series data, and its ability to be extended to deep learning (**right**). The two critical hyperparameters that need to be chosen correctly for NNET models are the embedding dimension, *m*, and the number of hidden units, *D*. For NNET models with a deep architecture (D-NNET), the number of hidden layers also needs to be determined.

Artificial neural networks are a type of artificial intelligence technology that mimics the human brain’s powerful ability to recognize patterns [39]. These models have been used successfully for modelling a broad range of time-series data [36,38,41,42]. The task of the artificial neural network is to model the underlying data-generating process during training so that valid forecasts can be made when the parameterized model is subsequently presented with new input data [35]. The most widely used and often preferred models when building artificial neural network forecasting models are those with a Multilayer Perceptron architecture, given its computational efficiency and efficacy and its ability to be extended to deep learning (Figure 1) [1,35,36,38,41,43,44]. Mathematically, a basic artificial neural network model (NNET) can be represented as follows:

(1) $$x_{t+s}=\beta_{0}+\sum_{j=1}^{D}\beta_{j}g(\gamma_{0j}+\sum_{i=1}^{m}\gamma_{ij}\;x_{t-\left(i-1\right)d})$$

in which there are two critical hyperparameters that need to be chosen, the embedding dimension, *m*, which captures the autocorrelation structure of the time series, and the number of hidden units, *D* [35,41,43]. For an artificial neural network model with a deep architecture (D-NNET), the number of hidden layers also needs to be determined [38,45].

Given that there is no general rule that can be followed to select the most appropriate hyperparameters, training data are used to estimate the optimal hyperparameter values and model architectures based on minimizing error-related terms during training. This step is typically computationally expensive. Using this approach, pretrained hyperparameter-optimized NNET models can be built. These models represent a simplified approach adept at handling tasks with constrained data availability, owing to their streamlined architecture and efficient training process. Yet, their simplicity can sometimes come at the expense of performance, as they are not able to extract deep features [46]. Conversely, D-NNET architectures describe models that utilize multiple hidden layers to represent features at higher and more abstract levels that are learned from the data [46]. D-NNET architectures, characterized by their increased complexity and capacity for performance, often demand substantial computational resources and extensive datasets for more effective training and deployment. However, the greater the number of weight parameters relative to the size of the training data, the greater the ability of the network to memorize the idiosyncrasies of individual observations. As a result, model generalization can be lost, leading to the development of a model that can be of little use in forecasting [39]. While the flexibility of artificial neural network models provides a potentially powerful forecasting tool, hyperparameter/parameter determination can complicate the design process [39].

### 3.2. Forecasting through Recurrent Topology

Forecasting through Recurrent Topology (FReT) is a time-series data prediction algorithm method that is based on learning recurrent patterns in a series’ local topology [38]. It is a versatile algorithm that reduces computational complexity and cost, with demonstrated feasibility using a variety of dynamic systems [38]. Unlike highly parameterized models, there is no need for computationally costly hyperparameter optimization and tuning procedures in high-dimensional parameter space.

FReT works by taking a data sequence where $\overset{⃑}{x}$ represents a one-dimensional time-series vector:

(2) $$\overset{⃑}{x}=(x_{1},x_{2},x_{3},\ldots ,x_{n})$$

and generates a Euclidean distance matrix (D), which is remapped to a local 3 × 3 neighbourhood topological matrix ($T^{\prime }$):

(3) $${\left(T_{ij}^{\prime }\right)}_{8}\;:D\to \;T^{\prime }$$

(4) $${\left(T_{ij}^{\prime }\right)}_{8}=\sum_{q=1}^{8}\;s(g_{q}-g_{0})2^{q-1};\;s\left(x\right)=\left\{\;\begin{array}{c} 0,\;x<0\\ 1,\;x\geq 0 \end{array}\right.$$

**Figure 2:**
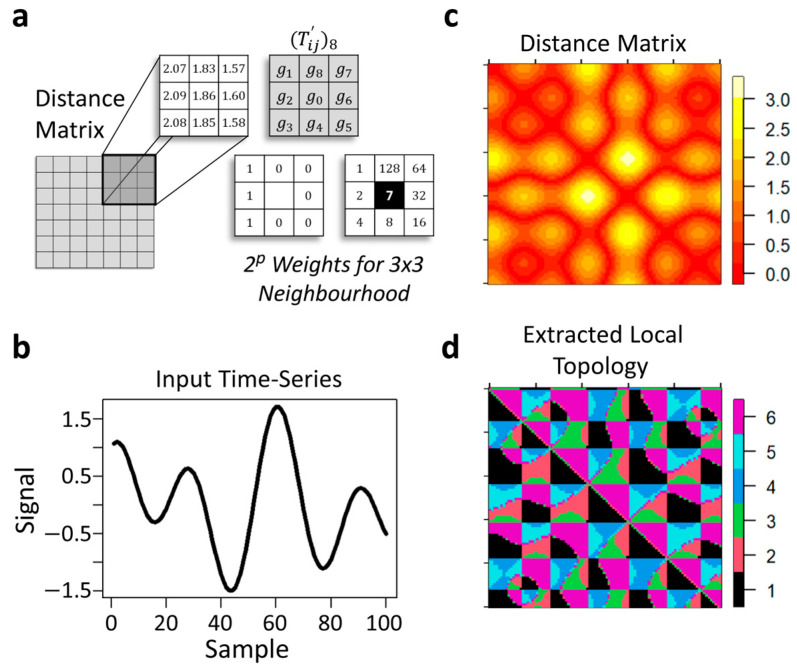
Extracting local topological patterning. A schematic illustrating the basic premise behind local topological pattern extraction (**a**). The process starts by creating a distance matrix from an input time-series signal. A binary code is then created by moving around the central point, *g*_0_, where a single integer value is calculated based on the sum of the binary code elements (0 or 1) multiplied by the eight positional weights. This is carried out over the entire distance matrix. (**b**–**d**) present an example of this process. A simulated time series (**b**), its traditional distance matrix (**c**), and the extracted local topological matrix (**d**). See text for additional details.

A binary code is then created by moving around the central point, *g*_0_, where a single integer value is calculated based on the sum of the binary code elements (0 or 1) multiplied by the eight positional weights (Figure 2a). This represents 8-bit binary coding, where there are $2^{8}\;$(256) different possible integer values that are partitioned into sextiles (six flattened layers) [38], generating a two-dimensional local topological matrix ($T^{\prime }$) composed of a set of six integer values, Z={1,2,3,4,5,6} (Figure 2b–d). Each point along the two-dimensional matrix diagonal represents a point in the signal sequence and its associated row vector (Figure 2d).

**Figure 3:**
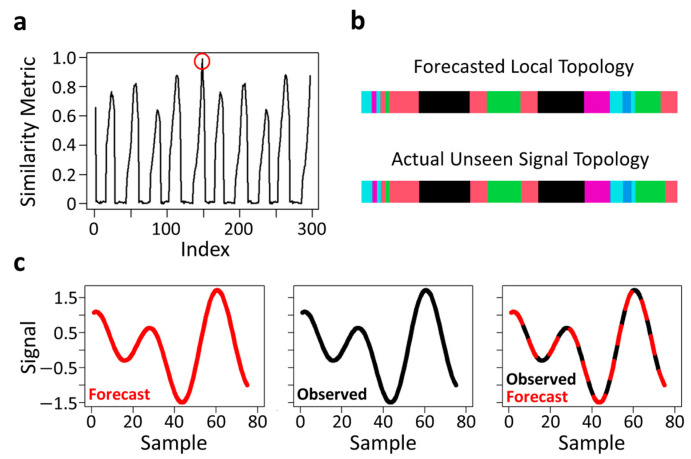
Topological patterning and archetype identification. A similarity metric related to the index of the training data that is used for topological archetype identification that effectively reduces to a simple maximization problem ((**a**), red circle). Panels b-c present the forecasted local topology of this identified archetype and the actual unseen signal topology of a simulated time-series signal ((**b**); very similar although not quite identical) that is used to construct an embodied model of the system’s upcoming dynamics (**c**). The colors in panel b reflect the set of six integer values.

From $T^{\prime }$, element-wise differences in all prior row vectors are computed with respect to the last row vector (an index of the system’s current state), generating a 1 (true) if their difference equals zero, otherwise 0 (false). These binary elements are summed and divided by the length of the row vector. This generates a similarity metric (Figure 3a) that ranges from 0 to 1 that can be used to find the index (+3 to account for the padded local topological matrix and beginning one step in the future) of the encoded training data topological archetype(s). This similarity metric differentially weights the importance of each part of the input training data. This produces a one-dimensional weight vector with respect to the system’s current state. The higher the values, the more closely the topological sequences align with the system’s current state based on topological patterning [38]. These topological archetypes ultimately reveal unique memory traces of past system behaviour(s) that can be used to construct a single multi-step-ahead embodied model of a dynamic system’s upcoming unseen dynamics [38] (Figure 3b,c). This approach has recently shown promise for forecasting human gait kinematics [38].

### 3.3. Kinematic Trajectory Forecasting

**Figure 4:**
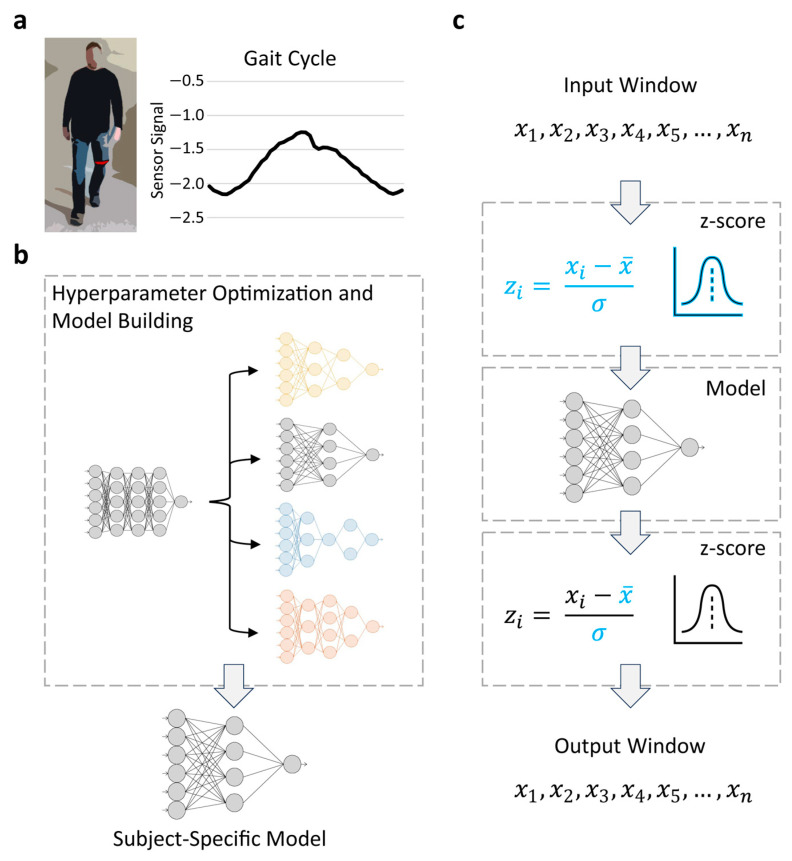
A schematic of the data processing workflow. Gait data were collected by a wearable sensor (illustrated in red in (**a**), left). The sensor is attached to the leg just above the patellofemoral joint line for recording gait kinematics based on hip flexion/extension using the Ambulosono system ((**a**), right). A subject’s training dataset was used to estimate hyperparameters and model architectures specific to an individual (subject-specific model) for NNET and D-NNET models (**b**). This was done on an external computer. For testing on the embedded system, the data for each subject’s input window were first *z*-score-scaled before being fed into their subject-specific model (**c**). The forecasted gait data, with the output as a *z*-score, were then converted back to the original sensor scale based on the attributes (mean and standard deviation) of the *z*-scored input window data. This allowed comparison to the true sensor signal test data (**c**).

An advantage of FReT is that its forecasts are based on the original data and associated scale [38]. Kinematic trajectory forecasts based on FReT can therefore be generated directly from the input window data. For NNET and D-NNET models, the training dataset was first used to estimate hyperparameters and model architectures before being deployed to the embedded system (Figure 4a,b). For testing, data were *z*-score-scaled to standardize model training, and the data of each input window were first *z*-score-scaled before being fed into the model (Figure 4c). The forecasted gait data, a *z*-score output, were then converted back to the original sensor scale based on the attributes (mean and standard deviation) of the *z*-scored input window data. This enabled comparison to the true sensor signal test data (Figure 4c).

### 3.4. Gait Sensor

Gait data were analyzed from a heterogeneous sample of fifteen (*n* = 15) adult participants without neurological abnormalities previously collected using the first-generation Ambulosono wearable sensor system [47]. The sensor system is based on using motion processor data consisting of a 3-axis Micro-Electro-Mechanical System (MEMS)-based gyroscope and a 3-axis accelerometer. The system and its firmware continuously record gait cycle dynamics and gait metrics while controlling for angular excursion and drift [47,48,49,50]. The sensor is attached to the leg just above the patellofemoral joint line through the use of a high-performance thigh band. This is the optimal location for recording gait features based on hip flexion/extension using this system [47,48,49,50].

### 3.5. Gait Data

The software application utilizes sensor data for step parameter calculations based on its ability to automatically detect hip flexion/extension in real time. The algorithm works by using a biomechanics model that uses circumference geometry and its concepts of radius and radians with limb length to functionally relate gait parameters to hip flexion/extension angle [47]. For this study, we used hip joint flexion/extension data, which are fundamental to this biomechanical model of gait. Hip joint flexion/extension data are also important for gait rehabilitation and human locomotion assistance [16]. While wearing the sensor, participants were asked to complete a 30 m walk at a self-selected pace [47]. Each participants’ time-series data (600 data points) were partitioned (300 data points), with the partitions being roughly split between training and testing sets.

### 3.6. Embedded System

**Figure 5:**
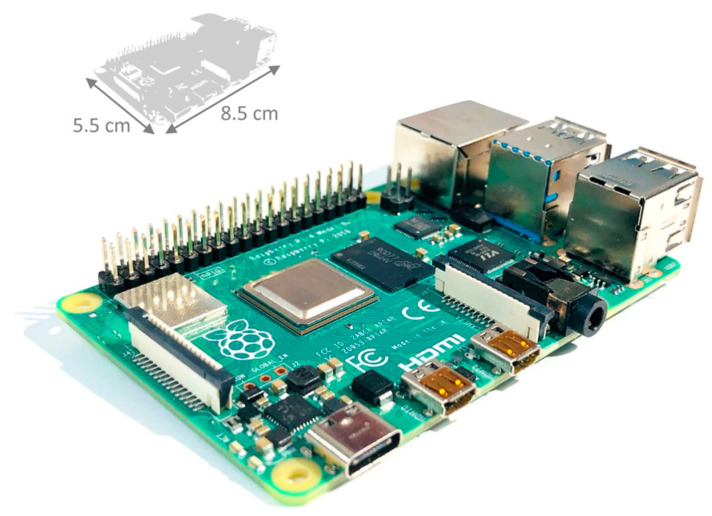
The embedded system that was prototyped. An illustration of the Raspberry Pi 4 and its dimensions (inset).

The embedded system that was prototyped was the Raspberry Pi 4 (https://www.raspberrypi.com/) (Figure 5). The Raspberry Pi is a very popular system that is affordable, well supported, and can run multiple code threads simultaneously. It is also suitable for performing multifaceted tasks, such as running complex control systems, and can be easily integrated with next-generation low-cost and low-power-consumption motion processors for future development. The Raspberry Pi, therefore, represents a valid prototyping base and highly versatile system that has the potential to be easily incorporated into lower-limb assistive ambulatory devices. In fact, the Raspberry Pi 4 was recently used as an embedded system to read sensor data and perform real-time exoskeleton control and optimization [26]. The Raspberry Pi 4 consists of a Broadcom BCM2711 Quad core Cortex-A72 (ARM v8) 64-bit SoC @1.5 GHz processor.

### 3.7. Model Deployment

Forecast model deployment was accomplished with R (https://www.R-project.org/) (accessed on 24 October 2023) by first installing Visual Studio Code on the Raspberry Pi, a popular source-code editor that can be used with a variety of programming languages, followed by the R extension. For NNET model implementation, the tsDyn package [43] was deployed. For D-NNET model implementation, the nnfor package [45] was deployed. FReT does not require any R user library packages.

### 3.8. Data Analysis

The models were evaluated by feeding the gait test set data into the embedded system, and the time to execute the forecast and the accuracy of the forecast were determined. For accuracy, we used the normalized root-mean-square error (NRMSE) as it facilitates comparisons between models by relating the error to the observed range of the data and simplifies the understanding of error rates for cross-disciplinary research [37]. The NRMSE is simply a standardized form of the commonly used root-mean-square error (RMSE) evaluation metric:

(5) $$\mathrm{RMSE}=\sqrt{\frac{\sum_{i=1}^{n}{\left(x_{i_{\mathrm{forecast}}}-x_{i_{\mathrm{observed}}}\right)}^{2}}{n}}$$

(6) $$\mathrm{NRMSE}=\frac{\mathrm{RMSE}}{x_{max}-x_{min}}$$

where $x_{max}$ and $x_{min}$ reflect the maximum and minimum values of the true sensor values that are being forecasted.

**Figure 6:**
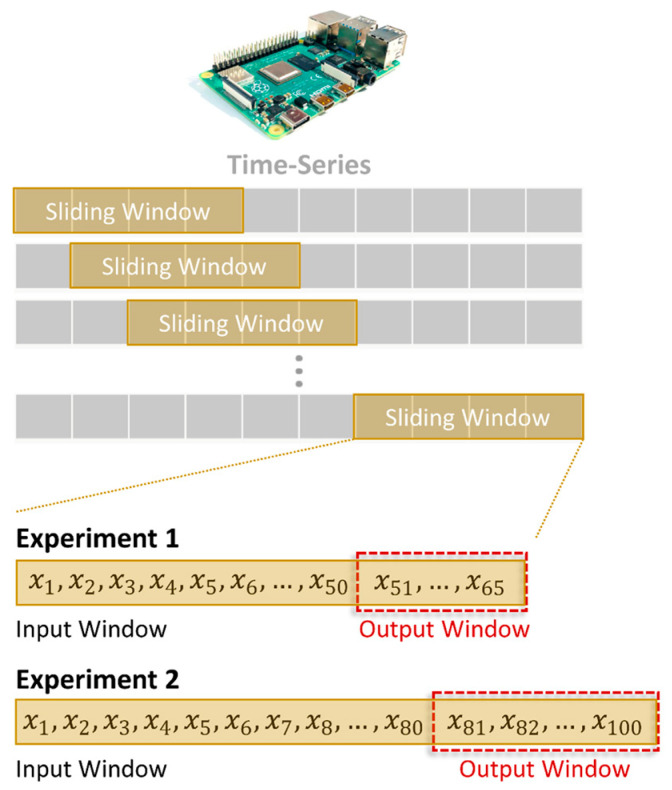
Different size input and output windows were evaluated (experiment 1 and experiment 2). Forecast accuracy and computational time per forecast (output window) for each individual were determined using a single-point sliding window.

Forecast accuracy and computational time per forecast for each individual were determined across the entire test set using a single-point sliding window (Figure 6). This approach uses new readings (input window) for each future gait trajectory forecast (output window) to avoid the accumulation of errors [1]. Different size input and output windows were also evaluated (Figure 6). For experiment 1, we tested a sliding input window of 50 data points and a forecast output window of 15 data points (approx. 400 ms) based on our previous work [38]. For experiment 2, we increased the testing sliding input window to 80 data points and used a prediction output window of 20 data points (approx. 550 ms). These forecast horizons are at least double that often investigated [1,6,37]. As artificial neural networks use random matrices, which can present problems as many perform well but others do not, we therefore also iterated this process 10 times to estimate the mean NRMSE and its dispersion (represented by the standard deviation of these 10 iterations). All analyses were carried out with R (R Core Team (2023). R: A Language and Environment for Statistical Computing. R Foundation for Statistical Computing, Vienna, Austria. https://www.R-project.org/).

## 4. Results

### 4.1. Forecasting Models

The NNET and D-NNET model architectures were coded in R [43,45] and estimated on an external computing source. The model hyperparameters were determined first via grid search to estimate an optimal value of hidden units, embedding dimension, and number of hidden layers (for D-NNET), similar to that carried out previously [1,38]. Once the model architectures were selected for each individual, these subject-specific hyperparameter-optimized NNET and D-NNET model architectures were deployed to the embedded system. FReT, which does not require hyperparameter optimization, was directly deployed to the embedded system for comparison with these pretrained hyperparameter-optimized NNET and D-NNET models.

**Figure 7:**
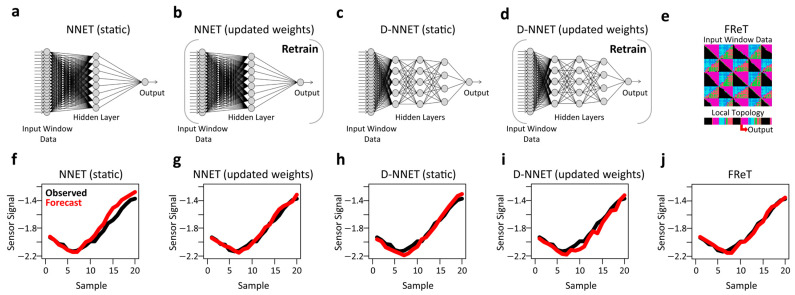
Comparison of models and their outputs. For all NNET and D-NNET models, hyperparameters were determined on an external computing source before being deployed to the Raspberry Pi. NNET and D-NNET models were run under two conditions. The first represents a static model where the model weights are fixed, and each sliding input window was simply fed into the fully pretrained model for predicting the unseen output window data (**a**,**c**). For the second condition, the model weights are updated by retaining with the input window data (**b**,**d**). Panel e illustrates topological archetype identification based on a simple maximization problem. (**f**–**j**) present example forecasted data with an input window of 80 data points and an output (forecast) window of 20 data points for each of the corresponding models shown above (**a**–**e**). Ground truth is shown in black, while each model’s predicted trajectory is shown in red.

The NNET and D-NNET models were each evaluated under two conditions. The first condition represents a static model. Here, the model weights are fixed, and each sliding input window was simply fed into the pretrained model for predicting the unseen output window data (Figure 7a,c). For the second condition, the updated NNET and D-NNET models (Figure 7b,d), the input window data were first used to retrain the model weights before predicting the unseen output window data. This approach provides a mechanism to update the model with real-time information embedded in the gait data signal that may not be appropriately modelled using the fully pretrained static models. FReT, by design, always updates based on the input window data, where the two-dimensional topological image reflects recurrent patterning in the gait signal’s surface topology (Figure 7e). Illustrative examples of forecasted output window kinematic gait trajectories for all the prediction approaches are also shown in Figure 7f–j.

### 4.2. Model Accuracy, Intra-Individual Variability, and Computational Time

Using the models presented in Figure 7a–e, we summarized the accuracy results across all the forecasting approaches for all fifteen participants (Table 1). The mean NRMSE is presented along with a measure of the mean intra-individual dispersion generated from 10 iterations per participant (see Materials and Methods). There, we can see that, unlike the network models, FReT exhibited greater precision (Table 1). Not only was FReT more consistent than the network models, but it was also more accurate than all the models tested (Table 1), with a NRMSE that was, on average, 46.2% lower across all the models/experiments.

**Figure 8:**
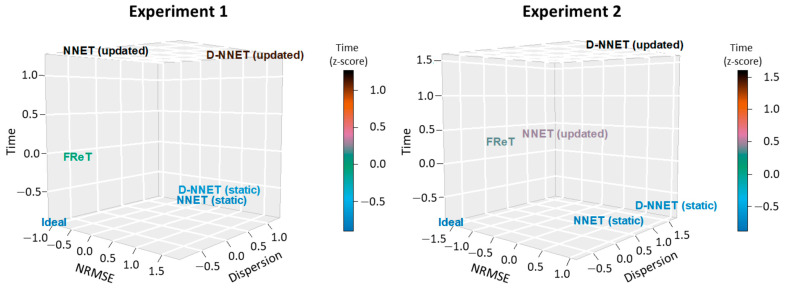
Model performance variables in multidimensional space. Performance variables for each model were *z*-score-scaled for plotting in multidimensional space. The bottom left corner (“Ideal”) represents the ideal condition of a perfect model with performance variables approaching zero.

Next, to ensure that FReT’s accuracy was not at the expense of computational cost, the computational time for each model was evaluated and is summarized in Table 2. While the static NNET and D-NNET models offered the best performance in terms of computational time (Table 2), they were not the most accurate (Table 1). The most accurate network model was the updated NNET model (Table 1). However, while the updated NNET model exhibited the best performance of all the artificial neural network models tested in terms of accuracy, FReT had less intra-individual variability, was more accurate, and was faster than the best-performing artificial neural network model (Table 1 and Table 2). Moreover, FReT also provided the best balance with respect to accuracy, intra-individual variability, and computational time when considering the combination of these performance variables. This is easily seen when plotted in scaled multidimensional space (Figure 8). There, it is evident that FReT tends to be located on the left, mid-to-lower side of the plot towards the ideal situation (i.e., the left bottom corner, which represents the idealized condition of a model’s performance variables approaching zero: zero error, zero computational time, and zero dispersion) (Figure 8). In fact, of all the models, FReT was closest to this idealized condition for both experiment 1 and experiment 2 (Figure 8 and Table 3).

### 4.3. Model Inter-Individual Variability

**Figure 9:**
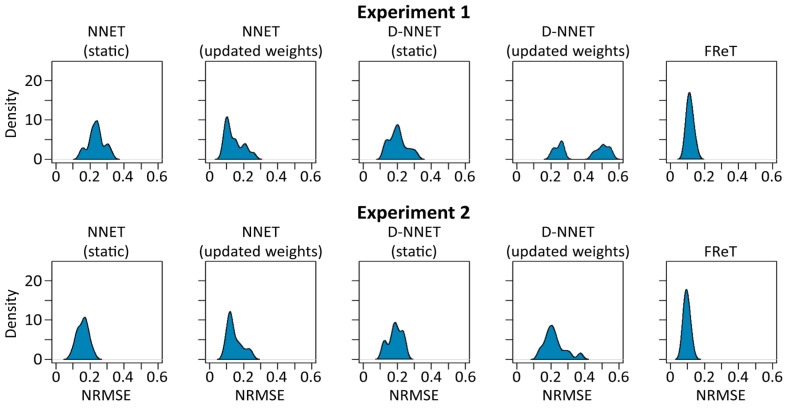
Distribution of NRMSE for FReT and the artificial neural network models. For each experiment, the distribution density of the average NRMSE per participant for all fifteen participants is shown for each model. The updated NNET model was the best-performing network model, while FReT was the best-performing model overall.

Our previous analyses focused on intra-individual model dispersion, which would tend to be more important for real-world applications as subject-specific training is preferable if the gait trajectory is to be used within a wearable device framework [6]. However, we also evaluated the dispersion of the average NRMSE per participant across all the participants to further showcase the utility of FReT. Dispersion in this sense represents inter-individual variability and can provide information regarding a model’s generalizability. The NRMSE distribution densities for all fifteen participants are shown for each model in Figure 9. There, it can be seen that FReT exhibited the highest density of all the models, with the peak of the light-tailed distribution density centered around the average NRMSE per participant (Figure 9). Together, these data, in addition to the accuracy, intra-individual variability, and computational time data, lend support to the idea that patterns in local topological recurrences embedded in wearable sensor data can be used to effectively forecast human gait kinematics.

## 5. Discussion

### 5.1. Complex Prediction Models

In recent years, artificial neural network models have attracted increasing attention with respect to time-series forecasting. It is well established that these models represent a versatile computational framework that can be used for modelling a broad range of time-series data [35,36,38,41,43], including gait trajectory prediction within the context of machine learning-enabled assistive ambulatory device design [1]. For example, using wearable sensor data, Su et al. proposed a gait trajectory prediction model that was based on a long short-term memory (LSTM) network with a weighted discount loss function [6]. This approach was, overall, able to predict the gait trajectory for multiple time frames, although the model was designed to more accurately predict the gait trajectory of the immediate future (e.g., 100 ms) [6]. Karakish et al. noted the limitations associated with computational load and relative inference time with the use of more complex models such as LSTM on embedded systems [1]. They decided to explore the use of simpler artificial neural network models, including D-NNET and convolutional neural network (CNN) models, on an embedded system to predict motion sensor data. Using these models, they were able to achieve comparable results, even after reducing the size of the networks and with forecast horizons of 200 ms [1]. However, it is important to point out that unlike FReT, all of these models still rely on neural network-based architectures and model hyperparameters that need to be chosen appropriately [1,6,35,36,38,41,43]. With these and many other types of complex forecasting models, there is no general rule that can be followed to select the most appropriate values. These values, therefore, need to be estimated based on available training data [41,43]. In fact, it has already been pointed out in the literature that this learning process, particularly for highly complex prediction models, needs to be improved, as it can take extended periods of time which may be unacceptable for time-series prediction in real-world applications [46]. Forecasting gait with FReT, on the other hand, avoids the need for the selection of hyperparameters and the uncertainty associated with their selection. In this study, we have also shown that FReT may offer a simple approach to predict gait kinematic trajectory at horizons > 200 ms. This study further highlights that more complex models are not always necessary for top predictive performance and can impose unnecessary computational load and power consumption constraints [38].

### 5.2. Embedded Systems

Within the framework of developing an embedded active assistive ambulatory system, it has already been suggested that it makes more sense to develop a system that uses wearable sensors for both the training and deployment stages rather than relying on a fixed-image capture system to record gait motion [1]. While models have been built based on fixed-motion capture systems (e.g., [37]), the uncertainty related to their transferability outside a fixed-motion capture environment has been less well studied. Moreover, problems can arise in situations where the motion capture markers used for gait are obscured by arm swinging or hand rails that may be needed for safety in certain clinical populations [51]. Unlike fixed-motion capture systems, embedded systems have numerous practical and important applications in many areas of rehabilitation science and biomedical engineering [1,6,9,17,18,19,20,21,22,23,24,25,28,29]. For example, as previously noted [37], the accurate sensor-aided prediction of gait kinematic trajectories can serve as a feedforward mechanism to powered devices instead of predominantly relying on feedback sensors, effectively serving to improve device performance by avoiding alterations in the user’s natural gait trajectories [37]. The implementation of FReT on embedded systems for sensor-aided gait kinematic trajectory prediction may also have several important operational advantages. First, being able to accurately predict gait time-series data while avoiding the drawbacks associated with modern prediction models, including random matrices and sensitivity to input hyperparameter selection, may help facilitate the design of a control system that works stably under a variety of conditions. Second, given that FReT does not require optimization and tuning techniques, it can be easily adjusted based on natural variations in walking speed. This is particularly important as it eliminates the need for pretraining data collection that is needed for commonly used machine learning models for model building. The requirement for collecting training data places an increased burden on both participants’ and clinicians’ time and is subject to several technical factors that may impact the quality of training data generalization. Finally, FReT does not require the time-consuming grid searches that are needed by other models during model building [1,6]. This can enable an adaptive, user-centric forecasting approach within an embedded framework that utilizes the fast arrival of new data for predicting upcoming gait dynamics.

### 5.3. Study Limitations

We acknowledge that there are several study limitations. First, the gait data for this study were collected at an individual’s preferred, self-selected walking speed. Data for variable walking speed were not evaluated, which is likely to reflect more dynamic, naturalistic environments. However, this may pose much less of a problem for FReT compared to many other types of complex forecasting models. In fact, several studies have found that commonly used prediction models for measures of gait perform much poorer when not trained on intra-subject data [1,6,34]. This can be problematic for traditional models requiring model building as the training data would need to reflect all potential environments, for all individuals, that may be encountered during deployment. FReT effectively avoids this issue as it was initially designed to enable CL-based forecasting on prior signal dynamics. Nevertheless, whether FReT can perform as well under dynamic walking conditions needs further testing. Second, this study also did not focus on clinical populations with variations in gait function, including individuals with walking disabilities and/or a history of falls. These would need to be evaluated to determine the applicability of this approach in a wider context and to understand its potential application to fall prevention. Finally, we did not evaluate the integration of multi-source information for motion intention [2,3]. However, FReT can be used for decoding multidimensional systems and predicting multidimensional signal topology [38].

## 6. Conclusions

The overall goal of this study was to deploy FReT on an embedded system to evaluate its ability to forecast wearable lower-limb motion sensor data. FReT was compared with several different artificial neural network and deep artificial neural network model architectures. This included models with both static model weight parameters and iteratively updated model weight parameters, with the latter serving as a mechanism to update the models with real-time information embedded in the gait signal that may not be appropriately modelled using fully pretrained static models. Our results indicate that FReT provided the best balance with respect to performance, more closely matching the idealized condition of a perfect model than all the other models evaluated. The proposed FReT framework has the advantage of being more accurate with a lower computational load and better consistency relative to the best-performing NNET model for predicting gait kinematic trajectories. Taken together, this study suggests that FReT can be used on an embedded system to effectively forecast gait cycle kinematics, supporting a new continuous learning capability that may work stably across a variety of conditions.

## Figures and Tables

**Table 1:** Accuracy comparison between models.

	Experiment 1		Experiment 2	
Model	NRMSE	Dispersion	NRMSE	Dispersion
NNET (static)	0.237	0.047	0.157	0.027
NNET (updated)	0.143	0.004	0.146	0.008
D-NNET (static)	0.203	0.055	0.188	0.054
D-NNET (updated)	0.535	0.023	0.221	0.014
FReT	0.115	0.000	0.097	0.000

Experiment 1: sliding input window of 50 data points and a prediction output window of 15 data points (approx. 400 ms). Experiment 2: sliding input window of 80 data points and a prediction output window of 20 data points (approx. 550 ms). NRMSE: normalized root-mean-square error. Dispersion represents the standard deviation of the mean NRMSE across 10 iterations.

**Table 2:** Comparison of computational time between models.

	Experiment 1	Experiment 2
Model	ms	ms
NNET (static)	9.534	11.11
NNET (updated)	195.0	218.8
D-NNET (static)	17.83	19.99
D-NNET (updated)	186.6	395.3
FReT	80.02	200.4

Experiment 1: sliding input window of 50 data points and a prediction output window of 15 data points (approx. 400 ms). Experiment 2: sliding input window of 80 data points and a prediction output window of 20 data points (approx. 550 ms).

**Table 3:** Distance between models and the idealized condition of a perfect model.

	Experiment 1	Experiment 2
Model	Euclidean Distance	Euclidean Distance
NNET (static)	2.32	2.40
NNET (updated)	2.33	2.36
D-NNET (static)	2.52	3.55
D-NNET (updated)	3.74	3.83
FReT	1.10	1.77

Euclidean distance between the idealized condition of a perfect model (i.e., zero error, zero intra-individual variability, and zero computational time) and the evaluated forecasting models.

## Data Availability

The data presented in this study are available from the corresponding author upon reasonable request.
